# Modifiable Risk Factors for Early Mortality on Hemodialysis

**DOI:** 10.1155/2012/435736

**Published:** 2012-07-24

**Authors:** Rory McQuillan, Lilyanna Trpeski, Stanley Fenton, Charmaine E. Lok

**Affiliations:** ^1^Division of Nephrology, Toronto General Hospital and The University of Toronto, 8NU-844, Toronto, ON, Canada M5G 2C4; ^2^The Renal Disease Registry, 585 University Avenue, Toronto, ON, Canada M5G 2C4

## Abstract

Data of incident hemodialysis patients from 2001 to 2007 were abstracted from The Renal Disease Registry (TRDR) from central Ontario, Canada and followed until December 2008 to determine 90-day mortality rates for incident hemodialysis patients. Modifiable risk factors of early mortality were determined by a Cox model. In total, 876 of 4807 incident patients died during their first year on dialysis; 304 (34.7%) deaths occurred within the first 90 days of dialysis initiation. The majority of deaths were attributed to a cardiovascular event or infection and more likely occurred in older patients and those with cardiovascular co-morbidities. Of potentially modifiable risk factors, low body mass index (<18.5), a surrogate for malnutrition, was a strong predictor of early mortality [adjusted hazard ratio (HR) 4.22 (CI: 3.12–5.17)]. Also, central venous catheter use was associated with a 2.40 fold increase risk of death (CI: 1.4–3.90). Patients who attended a multidisciplinary pre-dialysis clinic were less likely to die (HR: 0.60, CI: 0.47–0.78). The first 90 days after initiation of dialysis is a period of especially high risk of death. We have identified potentially modifiable risk factors in vascular access type, pre-dialysis care and nutritional status.

## 1. Introduction

End stage renal disease (ESRD) is increasing with over 400,000 patients in North America requiring dialysis [1]. Despite modern technology and medicines, mortality on dialysis continues to be high with an average 5-year survival of approximately 33% for patients with diabetes and 50% in patients without [1]. While multiple studies have examined associative factors for this long-term survival, less attention has been paid to very early survival on dialysis. Historically the United States Renal Data Services (USRDS) did not include patients in survival analyses until they had already completed 90 days on dialysis [2]. In order to allow for international comparisons other large international registries such as the UK Renal Registry and the Australia and New Zealand Dialysis and Transplant Registry (ANZDATA) adopted this convention in reporting survival [3, 4]. Consequently less is known about initial survival or the risk factors associated with early mortality in dialysis patients. However, mortality during the first 90 days remains high, and it is in this early critical period where modifications may be made which may impact not only early survival, but also possibly longer term survival on dialysis.

Knowing accurate rates and causes of mortality in this early 90 day initiation period may allow us to identify some modifiable risk factors for early mortality. Furthermore, information about the probability of survival may be of benefit to patients and physicians in making informed choices about starting dialysis. The aim of this study is to determine the incidence and risk factors for 90-day mortality in newly initiated hemodialysis patients from a large prospective, nationally representative cohort of patients followed by The Renal Disease Registry (TRDR). 

## 2. Methods

### 2.1. Data Source

All incident hemodialysis patients over a 7-year period from 1st January 2001 to 31st December 2007 were obtained from TRDR in Ontario, Canada. This registry covers a population base of the 7.6 million people in Central Ontario. There are 35 participating dialysis sites. Baseline demographic data such as age, gender, race, residential details, and comorbidities such as diabetes, hypertension, cardiovascular disease, pulmonary disease, peripheral vascular disease, and malignancy are collected. The primary renal diagnosis is recorded at the time of dialysis initiation. This incident data is submitted by the treating dialysis centre. The registry longitudinally follows patients and updates information on dialysis modality switches, renal transplantation, withdrawals from dialysis, and deaths on a monthly basis.

### 2.2. Analysis

All ESRD patients who initiated hemodialysis between 2001 and 2007 were included in the analysis. Patients were considered to have initiated dialysis once they were receiving out-patient hemodialysis. The primary outcome was death during the first 90 days of dialysis. 

The incidence of mortality in the first 90 days was determined by calculating the proportion of patients who died over the total number of patients who initiated dialysis. A Cox proportional model was used to determine risk factors for death in the first 90 days of the initiation of hemodialysis treatment. Hazard ratios (HR) and corresponding 95% confidence intervals (CI) were adjusted for case-mix differences in age, gender, race, body mass index (BMI), cause of ESRD, comorbidities (diabetes, cardiovascular disease, cerebrovascular disease, peripheral vascular, pulmonary disease, malignancy, anemia), initial type of vascular access,and predialysis care. Predialysis care was categorized as no predialysis care, patient followed by a nephrologist or seen in a multidisciplinary predialysis clinic. Patients who died after 90 days, received a transplant, transferred out of participating TRDR sites or were lost to follow up were censored at the event date or until the end of the study period, December 31, 2008. Analyses were performed using the SAS statistical software package version 9.1.3. (SAS, Inc., Cary, NC, USA).

## 3. Results

Four thousand eight hundred and seven patients started hemodialysis between 2001 and 2007. Characteristics of these patients are summarized in Table 1. The mean age ofpatients was 66.1 years; 59.9% were men and 69.4% were Caucasian. 

Among these new patients participating in TRDR over the 8-year study period, there were 304 deaths (6.3%) within the first 90 days of starting dialysis. In the first year of dialysis, 876 patients died. The death rate over the first year was not constant, and 34.7% percent of all deaths during the first year occurred in the first three months. 

Table 2 shows risk factors for mortality and their adjusted hazard ratios. Patients who died within 90 days tended to be older with those over the age of 75 years having more than 2.6 times the risk of dying as those under 65 years old. Of potentially modifiable risk factors, body mass index was a strong predictor of early mortality carrying an adjusted hazard ratio of 4.2 when BMI is less than 18.5. Ninety-three percent of patients who died started dialysis via a central venous catheter (CVC). Use of a CVC was associated with a 2.4-fold increase risk of death within 90 days. Patients who were seen in a multidisciplinary predialysis clinic were less likely to die (HR 0.6; CI: 0.47–0.78).

Table 3 shows the causes of death. A cardiovascular cause accounted for death in 34.2% of patients, with infection the next most common cause of death.

## 4. Discussion

The main finding of this study is that mortality during the first 90 days of initiating hemodialysis remains high, representing 34.7% of all deaths during the first year of dialysis. Not surprisingly, increasing age and cardiovascular disease are strong predictors of poor outcome; however, we also identified potentially modifiable factors in this early period of high mortality risk. The use of central venous catheters, low BMI (a surrogate for malnutrition), and lack of attendance at a nephrology clinic prior to commencing dialysis are strong and potentially modifiable independent predictors of early death on dialysis. 

In the United States, Medicare does not reimburse for patients on dialysis under the age of 65 years until they have completed 90 days on dialysis. In an effort to provide comparable analyses, the USRDS does not typically include any patients in their census until they have been on dialysis for this 90 day period. More recently, using Social Security Administration to fill in missing data from the first ESRD service date, survival data on the first 90 days has been made available by the USRDS. It has remained the convention, however, for international renal registries to report survival rates from 90 days onward. The more recent USRDS data suggests that mortality risk among incident dialysis patients seems highest soon after initiation of dialysis [5]. Our finding of 6.3% mortality in the first 90 days is consistent with these and with data published by the UK renal registry which reported that among 6634 incident patients requiring renal replacement, which included peritoneal dialysis and preemptive transplant patients, three hundred and eighty-six (5.8%) died within 90 days [6]. The Dialysis Outcome and Practice Study (DOPPS) showed that the risk of death for HD patients during the first year is at its highest during the initial 120 days. Among 4802 incident patients the risk of death was 27.5/100 person-years in the first 120 days versus 21.9/100 person-years for the remainder of the year [7].

When compared to over a decade ago, there appears to be no improvement in early mortality rates from prior studies. For example, based on data collected between 1990 and 1992 from multiple European centers, a 90-day mortality rate was found to be 3.9% [8], with marked variation between countries, ranging from 1.8% to 11.4%. This variation may reflect different reporting criteria, differing criteria for starting dialysis, or both. A US study of 15,245 dialysis patients for the same period found a 90-day death rate of 6% [9].

This apparent lack of improvement contradicts the expectations of advances in dialysis therapies and modern medicine. However this stagnancy may be accounted for, in part, by a change in practice towards accepting older, sicker patients onto dialysis programs. For example, the average age of our patients was 66.1 years, significantly older than both the DOPPS and ERA-EDTA studies at 57 and 54 years, respectively [7–9]. Those who survived to initiate dialysis may have also benefited from more comprehensive care of multidisciplinary CKD clinics [10–12] and may not have otherwise survived to initiate dialysis. Our data suggests that the specialty care offered in the setting of a multidisciplinary clinic offers survival advantage independent of vascular access formation, correction of anemia, and malnutrition (HR = 0.6; CI: 0.5–0.8). Such multidisciplinary care has previously demonstrated a reduction in urgent dialysis starts with fewer hospitalizations following dialysis initiation [11] and improved survival on dialysis [12]. Interestingly in this study a similar reduction was observed for patients seeing a nephrologist outside the context of a multidisciplinary clinic. Of note, multidisciplinary predialysis clinics at participating TRDR facilities are comprised of variable allied health care members, with varying degrees of nephrologist involvement. A potentially greater impact of multidisciplinary predialysis clinics may have been observed if multidisciplinary clinics were uniformly defined.

Despite the efforts of predialysis care, there are nonmodifiable risk factors for early mortality on dialysis. This information is useful on a practical level as it may inform the decision of the clinician, patient, and their family as to whether to initiate dialysis treatment and the likely course and outcome they may expect. For example, increasing age was associated with poor survival, with patients over 75 years of age representing 50% of patients who died within 90 days of starting dialysis. The presence of coronary artery disease, malignancy and chronic obstructive pulmonary disease were all associated with an increased risk of death. Clearly none of these factors are modifiable, but the insight of the additional risk of mortality may be useful in setting realistic expectations when initiating dialysis.

Malnutrition (BMI < 18.5) was found to be the strongest predictor of poor survival on dialysis with an adjusted hazard ratio of 4.2. This finding is in keeping with many other large studies, which have shown malnutrition assessed either by biochemical means or by measurement of BMI or anthropometric measurements to be associated with increased mortality [12–15]. There are a number of reasons why this may be so. Malnutrition in itself may increase susceptibility to infection [16]. Several features of malnutrition such as increased oxidative stress, increased plasma levels of fibrinogen, and inflammation may also increase the risk of cardiovascular disease [17]. It is debatable whether malnutrition is modifiable. Potential interventions include the professional input of a dietitian, oral or enteral nutritional supplementation, intradialytic parenteral nutrition, intraperitoneal nutrition, and appetite stimulants [18–20]. While some but not all studies have demonstrated an improvement in markers of nutritional status with these measures in the dialysis population, an improvement in outcome has never been demonstrated. It is likely that the benefit of improved nutrition would occur prior to dialysis initiation rather than following it. A large randomized control trial is needed to establish if improvement in predialysis nutritional status is associated with a reduction in early mortality on dialysis. 

This highlights the importance of well-planned and implemented predialysis nephrology care. Such care should promote the safe and optimal dialysis start of a patient: a well-educated and nourished patient starting dialysis as an outpatient with a fully matured arteriovenous fistula (AVF), ready for expert cannulation and function. It is well established that use of an AVF provides better quality of life, improved blood flows, results in fewer hospital admissions for access failure or related infections, and is associated with better survival and reduced healthcare costs than central venous catheters [21–24]. DOPPS data showed an increased relative risk of death of 1.31 for patients beginning dialysis via a central venous catheter. An even more striking increase in the risk of death associated with catheter use was noted in a recent USRDS study with the relative risk being 2.18 for catheter use compared to AVF use [22]. A large Canadian study demonstrated that incident catheter use was associated with a 6 times greater risk of death compared with fistula or graft use combined [25]. Despite these alarming statistics and the recommendations of the fistula first initiative [26], a significant proportion of ESRD patients begin dialysis using a CVC. The USRDS reported that 82% of patients initiated dialysis with a catheter in 2006 [27]. In our study, 79.9% of incident hemodialysis patients were dialyzed via a CVC and this was found to be both a clinically and statistically significant variable associated with early mortality (HR 3.4). Even after adjustment for age, comorbidity and late referral, this still conferred an almost 2.5 time risk of death within 90 days of starting dialysis. This study emphasizes the critical importance of timely creation of a functional AVF that is suitable for dialysis, which may turn out to be the single most modifiable intervention in improving early survival amongst dialysis patients.

The key strength of this study is that patients were identified at initiation of chronic hemodialysis. The data was gathered prospectively and reliable information regarding patient demographics and comorbidities was available. The study included over 4,800 incident patients starting hemodialysis between January 1, 2001 and December 31, 2007, who were then followed until December 31, 2008. Patients from 35 centers were included, thereby creating a nationally representative sample and ensuring applicability of the conclusions drawn. Limitations of the study include the lack of criteria for initiating chronic dialysis; this was up to the discretion of the treating nephrologist, as is practiced in most centers. So as not to distort the data by inclusion of cases of acute renal failure, patients who started dialysis while in hospital were not considered to be on the chronic dialysis program until after discharge from hospital. Thus, a patient with established advanced CKD who initiated dialysis during a hospital admission for an intercurrent illness would not have been included in our analyses if they died before discharge. Therefore our study may underestimate the true incidence of ESRD and the short-term mortality associated with starting dialysis.

## 5. Conclusion

Early 90-day mortality after initiation of dialysis remains high. In order to improve outcomes, the first necessary step is to ensure accurate reporting during this period. Early referral and attendance of predialysis clinics may facilitate the timely creation of functional AV fistula and correction of malnutrition, modifiable factors that may improve early survival on dialysis.

## Figures and Tables

**Table 1:** Patient characteristics for TRDR cohort 2001 to 2007.

Characteristic		Patients who died in 90 days (*n* = 304)		Patients who survived (*n* = 4503)		All (*n* = 4807)	
		*N*	%	*N*	%	*N*	%
Age (years)	<18	0	0	4	100	4	0.1
	18–44	10	1.9	503	98.1	513	10.7
	45–64	45	3.2	1379	96.8	1424	29.5
	65–74	96	7.7	1156	92.3	1252	26.1
	75–84	114	8.7	1203	91.3	1317	27.4
	85+	39	13.1	258	86.9	297	6.2

	Diabetes	110	5.8	1776	94.2	1886	39.2
Etiology ESRD	Renovascular	63	6.1	965	93.9	1028	21.4
	Other	131	6.9	1762	93.0	1893	39.4

	Diabetes	142	6.1	2176	93.9	2318	48.3
	Cardiovascular disease	186	8.5	2007	91.5	2193	45.6
Comorbidities	Peripheral vascular disease	83	9.3	814	90.7	897	18.7
	Cerebrovascular	66	9.7	616	90.3	682	14.2
	Malignancy	78	11.4	605	88.6	683	14.2

Sex	Male	195	6.8	2684	93.2	2879	59.9
	Female	109	5.6	1819	94.4	1928	40.1

	Caucasian	250	7.5	3087	92.5	3337	69.4
	Asian	22	5.2	401	94.8	423	8.8
Ethnic origin	Black	6	1.5	404	98.5	410	8.6
	Indian	15	4.2	342	95.8	357	7.4
	Other	10	3.6	270	96.4	280	5.8

Hemoglobin	<100 g/L	198	7.2	2566	92.8	2764	57.5
	>100 g/L	106	5.2	1937	94.8	2043	42.6

	Not seen	143	9.6	1343	90.4	1486	30.9
Predialysis	Seen by nephrologist only	25	4.6	517	95.4	542	11.3
	Seen in multidisciplinary clinic	135	4.9	2643	95.1	2778	57.8

	<18.5	79	19.9	318	80.1	397	8.3
BMI	18.5–30	171	5.3	3162	94.7	3333	69.7
	>30	53	5.0	998	95.0	1051	22.0

	Catheter	282	7.3	3558	92.7	3840	80.1
Access	Fistula	20	2.4	832	97.6	852	17.8
	Graft	2	1.9	102	98.1	104	2.1

**Table 2:** Risk factors for mortality.

Characteristic		Adjusted hazard ratio	C.I.	Unadjusted hazard ratio	C.I.
Age (years)	65–74	2.25	1.75–3.17	2.75	1.98–3.83
	>75	2.62	1.89–3.65	3.41	2.50–4.60

Sex	Female	0.74	0.59–0.95	0.83	0.66–1.05

Ethnicity	Asian	0.69	0.44–1.08	0.68	0.44–1.06
	Black	0.28	0.12–0.63	0.19	0.09–0.43
	Indian	0.74	0.43–1.26	0.56	0.33–0.94
	Other	0.6	0.33–1.11	0.51	0.28–0.94

Comorbidities	Diabetes	1.17	0.92–1.50	0.93	0.74–1.17
	Cardiovascular disease	1.44	1.12–1.60	1.90	1.51–2.40
	Peripheral vascular disease	1.34	1.02–1.77	1.64	1.28–2.12
	Cerebrovascular disease	1.34	1.01–1.79	1.70	1.30–2.24
	COPD	0.87	0.62–1.63	1.50	1.08–2.08
	Malignancy	1.72	1.32–2.25	2.14	1.65–3.76

BMI	<18.5	4.22	3.12–5.17	4.24	3.16–5.71
	24.9–30	1.05	0.70–1.44	0.99	0.70–1.53
	>30	1.17	0.83–1.66	0.98	0.70–1.33

Vascular access	Catheter	2.40	1.40–3.90	3.37	2.12–5.37
	Graft	0.98	0.23–4.23	0.85	0.20–3.67

Hemoglobin	<100 g/L	1.21	0.95–1.55	1.38	1.09–1.75

Predialysis care	Seen by nephrologist only	0.59	0.39–0.91	0.46	0.30–0.71
	Seen in multidisciplinary clinic	0.6	0.47–0.78	0.49	0.39–0.62

**Table 3:** Cause of death.

Cause of death	*N*	%
Cardiovascular	104	34.2
Infections	42	13.8
Malignancy	30	9.9
At home	40	13.2
Miscellaneous	49	16.1
Withdrawal	39	12.8
